# Analysis on the hidden cost of prefabricated buildings based on FISM-BN

**DOI:** 10.1371/journal.pone.0252138

**Published:** 2021-06-03

**Authors:** Junlong Peng, Jing Zhou, Fanyi Meng, Yan Yu

**Affiliations:** 1 College of Transportation Engineering, Changsha University of Science and Technology, Changsha, Hunan, China; 2 College of Civil and Transportation Engineering, Shenzhen University, Shenzhen, Guangzhou, China; University of Defence in Belgrade, SERBIA

## Abstract

Facing the pressure of environment, sustainable development is the demand of the current construction industry development. Prefabricated construction technologies has been actively promoted in China. Cost has always been one of the important factors in the development of prefabricated buildings. The hidden cost of prefabricated buildings has a great impact on the total cost of the project, and it exists in the whole process of building construction. In this paper innovatively studies the cost of prefabricated buildings from the perspective of hidden cost. In order to analysis the hidden cost of prefabricated buildings, the influencing factor index system in terms of design, management, technology, policy and environment has been established, which includes 13 factors in total. And the hidden cost analysis model has been proposed based on FISM-BN, this model combines fuzzy interpretive structure model(FISM) with Bayesian network(BN). This model can comprehensively analyze the hidden cost through the combination of qualitative and quantitative methods. And the analysis process is dynamic, not fixed at a certain point in time to analyze the cost. We can get the internal logical relationship among the influencing factors of the hidden cost, and present it in the form of intuitive chart by FISM-BN. Furthermore the model could not only predict the probability of the hidden cost of prefabricated buildings and realize in-time control through causal reasoning, but also predict the posterior probability of other influencing factors through diagnostic reasoning when the hidden cost occurs and find out the key factors that lead to the hidden cost. Then the final influencing factors are determined after one by one check. Finally, the model is demonstrated on the hidden cost analysis of prefabricated buildings the probability of recessive cost is 26%. In the analysis and control of the hidden cost of prefabricated buildings, scientific and effective decision-making and reference opinions are provided for managers.

## 1. Introduction

With the acceleration of China’s urbanization process, the traditional cast-in-situ concrete construction method has low production efficiency, high consumption of raw materials, serious environmental pollution, long construction period, large building energy consumption and more construction waste [1–3]. This is unable to meet the needs of sustainable development of the construction industry.

Prefabricated buildings is also called modular buildings [4]. Compared with traditional cast-in-situ concrete building, it can minimize construction time, save construction water and reduce environmental pollution [5–8]. In addition, the United Nations Environment Program (UNEP) points out that prefabricated buildings can also reduce building energy consumption and greenhouse gas emissions [9, 10]. But the cost problem has always restricted the development of prefabricated buildings [11, 12]. In the construction process, improper cost management can easily cause cost overruns and delays in construction period [13, 14]. The cost of prefabricated buildings is affected by many factors, and the cost management is a systematic, complex and dynamic process [15, 16]. Therefore, how to reasonably and effectively analyze and control the cost of prefabricated buildings becomes a necessary prerequisite for the development of prefabricated buildings.

The cost of construction project consists of explicit cost and hidden cost [17]. Explicit cost refers to the cost that can be quantified and directly expended in the process of project construction, such as labor cost, material cost, machinery cost, etc. At present, there are some studies on the hidden cost of construction projects, but there is no accurate definition of the hidden cost in the academic community [18, 19]. From the perspective of project management, the hidden cost refers to the opportunity cost in the optimal management mode, which can enable the resource organization to achieve excellent production capacity [20, 21]. Hidden cost is difficult to quantify, such as: Emergency of force majeure, professional level of managers, quality standards and so on.

In recent years, many scholars have discussed and studied the cost of prefabricated buildings from different angles and levels. Chen et al. [22] proposed that the construction cost of prefabricated buildings construction was 10%-20% higher than that of traditional cast-in-place construction. Arashbour et al. [23] through data investigation and simulation experiment, found that the installation cost, production cost and transportation cost of prefabricated components were the key reasons for the high cost of prefabricated buildings [24, 25]. Wang et al. [26] proposed a calculation model to estimate the total cost of prefabricated construction supply chain on the basis of activity-based costing (ABC). Bortolini R et al. [27] used the whole element dynamic monitoring and control technology of BIM 4D modeling technology to monitor the prefabricated buildings in real time to minimize the cost and maximize the benefits of resource allocation. There were also some researches that predicted and analyzed the cost of prefabricated buildings through methods such as back propagation (BP) neural network, gray system theory, and related algorithms combined with Matlab [28–31]. In general, most studies on the cost of prefabricated buildings mainly analyze the explicit costs, and few scholars analyze the hidden cost. However, the hidden cost has a great impact on the total cost of the project, with strong concealment and uncertainty, which is difficult to quantify, and there is a complex relationship between the hidden cost [21, 32]. Therefore, it is necessary to focus on the hidden cost of prefabricated buildings.

The cost control of construction project is composed of two parts: explicit cost control and hidden cost control. At present, the explicit cost has been relatively transparent in the construction project. In the industry, the research on explicit cost has been more in-depth and comprehensive, and the research on its management theory, method, calculation model and control measures have been also relatively mature. Therefore, the compressible space of explicit cost is small. However, due to the hidden and difficult to quantify the characteristics of hidden cost, managers generally do not pay much attention to the management, they will not take substantive measures until the project cost is out of control [19, 33, 34]. The control of hidden cost is the key to reduce cost and improve profit of construction project. It is an important way for construction enterprises to increase revenue and reduce expenditure, improve labor productivity and expand economic benefits, and it is also the key to enhance the internal core competitiveness. The research on the hidden cost of construction project has become a hot, difficult and important problem to be solved in the academic circles and enterprises. Therefore, compared with the explicit cost, it is more promising to put insights on the hidden cost, and this is of great significance to the cost control of prefabricated buildings.

At present, the research of prefabricated buildings cost has been mainly from the perspective of explicit cost. On the one hand, it has been qualitative analysis of the relationship between cost factors, on the other hand, it has been quantitative comparison with traditional cast-in-situ concrete buildings cost increment. The analysis angle of hidden cost has been innovatively used in this article. And qualitative and quantitative analysis have been combined to analyze the hidden cost of prefabricated buildings systematically and dynamically.

We combine FISM with BN from the perspective of construction side of employer in this study. Firstly, the hidden cost index system of prefabricated buildings is constructed from five dimensions of design, management, technology, policy and environment. Then, the appropriate BN of the hidden cost of prefabricated buildings is obtained by using FISM and causality diagram. Finally, through BN model learning and reasoning calculation, we can predict the probability of the occurrence of hidden cost, find out the key factors leading to the occurrence of hidden cost, which can effectively predict the hidden cost in advance. It provides an effective and feasible new way to analyze and manage the hidden cost of prefabricated buildings. This study can not only reveal the internal logical relationship between the hidden cost factors of prefabricated buildings, but also conduct real-time management analysis on the occurrence probability of hidden cost and the factors leading to the occurrence of hidden cost. In this way, the total project cost can be indirectly controlled. This is of great significance to the development of prefabricated construction industry and the cost management of practical projects.

## 2. Factor selection

### 2.1 Hidden cost of construction project

In the construction project cost management, the hidden cost and the explicit cost are relative, the hidden cost is hidden in the total project cost. At present, there are some studies on the hidden cost of construction projects. But the definition of hidden cost is not clear [18, 19]. Based on the related research results of hidden cost, the hidden cost can be roughly divided into two categories. One is from the perspective of the enterprises, the hidden cost of construction projects is the opportunity cost of enterprises. The other is from the perspective of project management, assuming that an optimal management mode can make the resource organization form achieve excellent production capacity, then the opportunity cost of the management mode in this state is the hidden cost of the project. The hidden cost in this paper is based on the second type. In the actual construction project, due to the continuous change of the project environment, the management mode also changes, so the hidden cost is uncertain. In the process of project implementation, hidden cost management is generally ignored by managers. Therefore, increasing the analysis and control of the hidden cost of the project is the necessary guarantee to make the total cost of the project reach the optimal.

### 2.2 Factor identification

**Fig 1:**
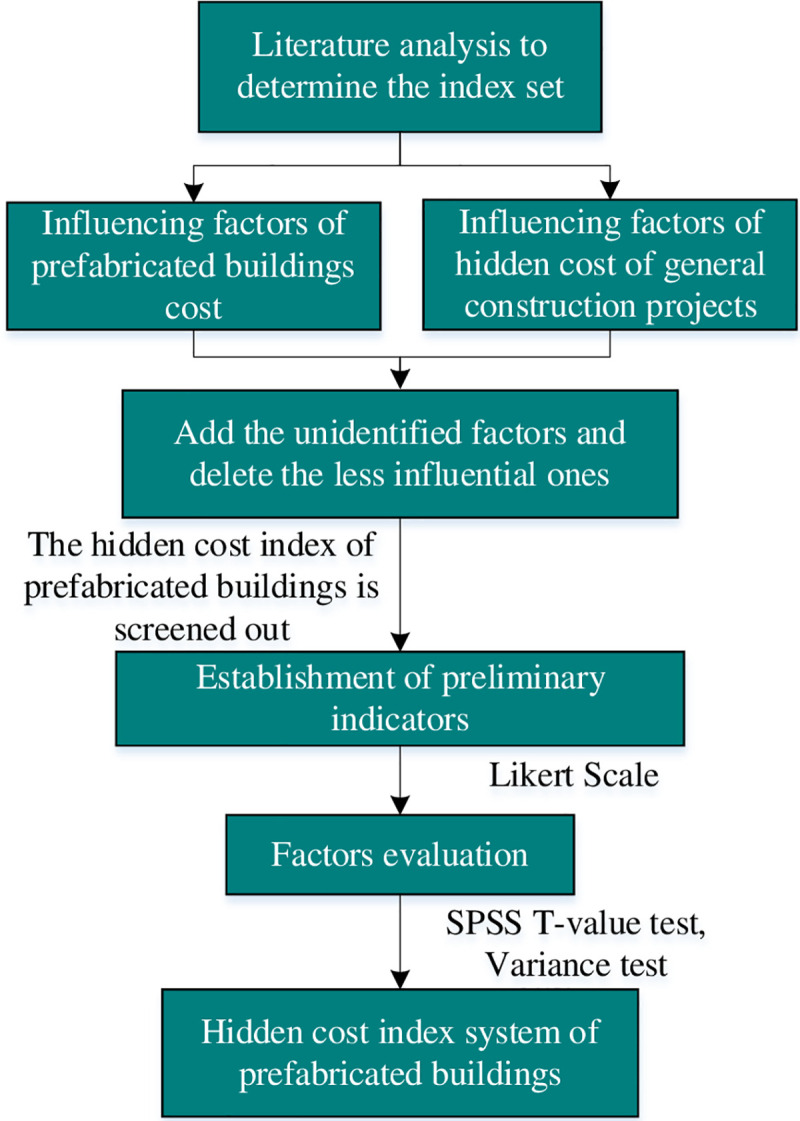
Index system establishment process.

In previous studies, there have been many discussions on the classification and selection of the influencing factors of the hidden cost of general construction projects, but there are few researches on the influencing factors of the hidden cost of prefabricated buildings. Due to the interoperability of construction projects, some hidden cost influencing factors of general construction projects are also the influencing factors of prefabricated buildings cost. Therefore, the combination of the two is analyzed to retain the hidden cost factors that influence the cost of prefabricated buildings in general construction projects. The specific process is shown in Fig 1.

In this paper, the key words of "hidden cost of construction", "hidden cost of construction" and "prefabricated buildings cost" were searched in Web of science, Google scholar, CNKI and other databases. Finally, 20 representative literatures were selected for analysis. From the design, management, technology, policy, environment five dimensions to statistical impact factors. Deleting the less influential factors and increasing the unidentified factors. This preliminary obtained the influencing factors of prefabricated buildings cost. And then, 16 influencing factors of the hidden cost of prefabricated buildings were selected. The form of on-site questionnaires, online answers, and Likert 5 scale were used. The respondents scored 16 factors according to the importance of 1~5. A total of 150 questionnaires were distributed, and 97 questionnaires were actually recovered, of which 61 were valid, and the effective recovery rate was 62.89%. Through the data analysis on SPSS 25.0, the reliability test and index screening of the questionnaire were completed. The reliability test results are shown in Table 1. According to Table 1, the reliability coefficient *α* = 0.875>0.8, indicating that the internal consistency of the data is high.

**Table 1:** The reliability statistics.

Reliability Statistics		
Cronbach’s Alpha	Cronbach’s Alpha based on standardization term	Number of items
0.874	0.890	16

The indexes were further screened by SPSS 25.0, and T-value test and variance test were used. In this paper, significance level *α* = 0.05 is selected, so the acceptance domain of hypothesis *H*0 was {*t*|*t*<*t*_0.95_(*n*−1)}, *n* = 61, and *t*_0.95_(60) = 1.671 by looking up the table (the relevant data was included in the S6 File). First of all, T-value test (2-tailed) is used to get that the site selection of prefabrication plant in design factors and the integrity of industrial chain in management factors do not meet the T-value test and should be eliminated. For the variance test, the greater the variance is, the greater the dispersion degree of the data is. When the variance value of a certain factor is greater than 1, the inconsistencies in the opinions of the surveyors on this factor are large, and this index should be eliminated. Then, through the variance test, the variance values of the engineering construction standard, the site selection of the prefabrication plant in the design factor and the integrity of the industrial chain in the management factor were all greater than 1, so these three factors were eliminated. Finally, the selection of prefabrication plant site, the integrity of industrial chain and engineering construction standards were excluded from 16 influencing factors, and the index system of influencing factors of hidden cost of prefabricated buildings is finally obtained, as shown in Table 2.

**Table 2:** Influencing factors of hidden cost of prefabricated buildings.

Target layer	Primary Indicators	Secondary Indicators
The Hidden Cost of prefabricated buildings (S14)	Design Factors (X1)	Rationality of splitting prefabricated components (S1)
		Selection of mechanical equipment (S2)
		Prefabrication rate and assembly rate (S3)
	Management Factors (X2)	Management experience and system (S4)
		Construction management system (S5)
		Resource allocation efficiency (S6)
	Technology Factors (X3)	Maturity of design system (S7)
		Component standardization and integration (S8)
		Technical level of professionals (S9)
	Policy Factors (X4)	National construction standards (S10)
		Tax policy (S11)
	Environment Factors (X5)	Emergency of force majeure (S12)
		Environmental restoration (S13)

### 2.3 Explanation of influencing factors

1. Rationality of splitting prefabricated components (S1): Unreasonable disassembly of prefabricated components will increase the difficulty of transportation and hoisting, and lead to the increase of production costs.
2. Selection of mechanical equipment (S2): The unreasonable selection of mechanical equipment for the production of prefabricated components will cause the failure to complete the prefabricated components on schedule. This will not meet the construction period of the project and eventually lead to the increase of project cost.
3. Prefabrication rate and assembly rate (S3): The effect of prefabrication rate on the increase of cost is different. Higher prefabrication rate will lead to shorter construction period and higher project cost.
4. Management experience and system (S4): Lack of project management experience and incomplete management system will not guarantee the normal progress of project construction, and eventually lead to the increase of construction cost.
5. Construction management system (S5): Scientific and reasonable construction management system are the premise of successful project completion.
6. Resource allocation efficiency (S6): Reasonable allocation of resources can improve production efficiency and reduce costs.
7. Maturity of design system (S7): The design of building type and how to solve the secondary splitting of components will affect the production cost of components.
8. Component standardization and integration (S8): The integration of unified component module is to make it universal and exchangeable, thus the design and construction efficiency are improved, and the cost is reduced.
9. Technical level of professionals (S9): Whether the professionals can make the project construction reach the optimal level will have an impact on the project cost.
10. National construction standards (S10): If the building does not meet the national construction standards, it will cause great economic loss, reputation loss and material waste.
11. Tax policy (S11): The tax rate of prefabricated components is higher than that of cast-in-place construction. The change of tax policy will affect the final profit of enterprises.
12. Emergency of force majeure (S12): When the construction is affected by force majeure, it will cause huge economic losses and even lead to casualties.
13. Environmental restoration (S13): If the treatment of environmental problems does not meet the requirements in the construction process, it will be punished, resulting in cost increase and construction period extension.

## 3. Research methods

Although the existing neural network, Adaptive-Network-based Fuzzy Inference Systems (ANFIS), rough sets, etc are widely used in project cost analysis [35–37]. Using these methods to analyze the project cost requires high sample data (For example, more data is needed, the data is a certain value, some model applications need to assume that the data has a certain linear relationship) [38–41]. And most of the studies are in a fixed time point on the quantitative analysis of cost, without the combination of qualitative and quantitative dynamic analysis.

Because of the single model is difficult to overcome the problem of hidden cost, which is uncertainty, the data is difficult to obtain a large number, and there is a complex relationship between hidden cost factors.

However, BN model shows good performance in data processing, and has no requirements for sample size and information integrity [42, 43], and has a mature foundation in dealing with uncertainty problems. At the same time, FISM can transform fuzzy concepts and views into a visual graph model with good structural relationship [44, 45], which can intuitively express the relationship between the influencing factors. Therefore, FISM and BN are combined to analyze the hidden cost of prefabricated buildings.

The FISM-BN hidden cost analysis model of prefabricated buildings constructed in this paper not only has no requirement on the size of sample data, but also can deal with the data with great uncertainty. It can also qualitatively reflect the complex relationship between factors in the form of intuitive graph, and can quantitatively and real-time analyze and predict the probability of hidden cost, which is dynamic. Therefore, FISM-BN is more suitable for analyzing the hidden cost of prefabricated buildings than the existing AI and heuristic algorithms.

The data in this paper has been collected anonymously. The data of questionnaire and expert interview have obtained the written consent of the participants. Before the respondents fill in the questionnaire, they will be informed in advance that the questionnaire will be used in the paper research. If they agree, they will fill in the questionnaire. If they disagree, they will not fill in the questionnaire. There is no data controversy. The questionnaire file consent is in the (S1 File–S4 File).

When interviewing experts, an interview consent will be issued in advance. In the interview consent, it will be clearly pointed out that the results of this interview will be used in the paper research. If they agree, they will be interviewed. If they disagree, they will refuse the interview. The interview consent is in the manuscript or the supporting information uploaded by the system.

### 3.1 The process of model establishment

**Fig 2:**
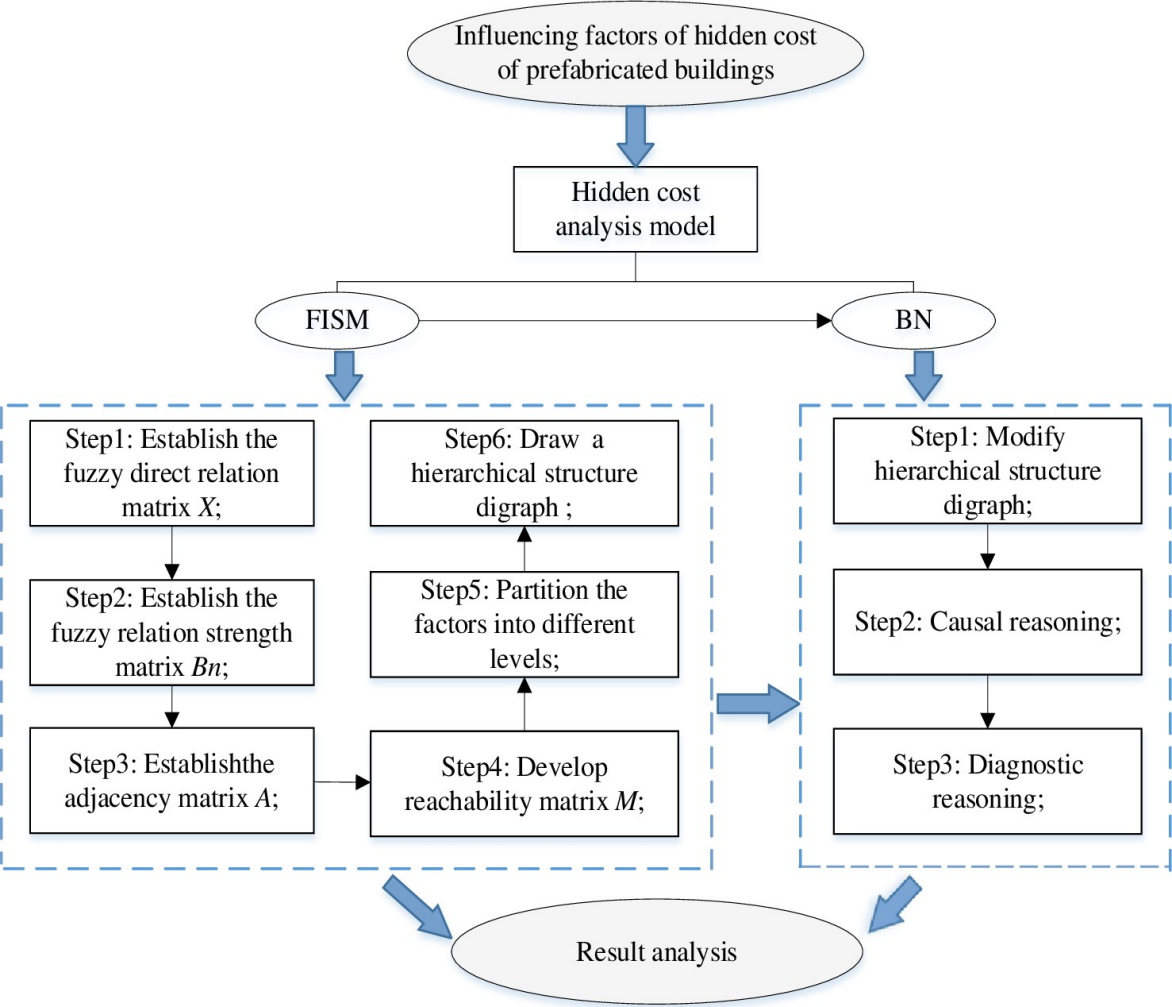
The process of FISM-BN hidden cost analysis model.

FISM and BN are combined to analyze the influencing factors. The hidden cost analysis model of FISM-BN is constructed. The specific process is shown in Fig 2. First of all, the main factors affecting the hidden cost were obtained. Then, the influencing factors were analyzed by FISM. In the FISM analysis, the first step is to establish the fuzzy direct relation matrix *X* to determine the mutual relationship between the factors. Secondly, according to the membership function, the fuzzy relation strength matrix *B*_*n*_ was obtained. The third step is to get the adjacency matrix *A* by selecting threshold *λ*. The fourth step is to get the reachable matrix *M* through Matlab. The fifth step is to partition the factors into different levels. The sixth step is to draw the hierarchical structure diagram of the influencing factors of the hidden cost of prefabricated buildings. In the process of FISM analysis, the relationship between the influencing factors could be obtained, and the influencing factors could be divided into surface direct factors, middle dynamic layer factors and deep guidance factors. Finally, the hierarchical structure chart was modified by the causality diagram method to get the appropriate BN structure of the hidden cost of prefabricated buildings. Then through the causal reasoning and diagnostic reasoning of BN, the probability of hidden cost occurrence and the factors leading to its occurrence are analyzed. and the probability of the influencing factors are sorted, and the relevant suggestions of hidden cost management are given.

### 3.2 Fuzzy interpretive structural model

The interpretative structural modeling (ISM) method was first introduced by Warfield (1974) and further developed by the Vanderbilt Columbus Laboratory in the U.S. ISM is used to analyze the problems related to the complex system structure [46, 47]. Fuzzy interpretive structural model (FISM) is an improvement on the basis of interpretive structural model [48], which introduces fuzzy mathematics into ISM. FISM can avoid the subjectivity of expert scoring to a certain extent, and make the analysis results more accurate and reasonable. By combining FISM with Matlab to deal with the relationship between factors, a clear hierarchical structure and hierarchical structure digraph will be obtained. It is more suitable for the system analysis with many factors, complex relationship and fuzzy structure.

① Establish *X*. The relationship between the two factors is preliminarily determined through expert interview. *X* = (*x*_*ij*_)_*n*×*n*_, where, *x*_*ij*_ is the correlation strength of factor *i* to factor *j*.

② Select membership function. According to Eq (1), the fuzzy relation strength matrix of influencing factors of prefabricated buildings cost is established, *B*_*n*_ = [*b*_*ij*_]_*n*×*n*_.


$$b_{ij}=\left\{ \begin{array}{l} x_{ij}/(x_{i}+x_{j}-x_{ij})\;i\neq j\\ 0\;i=j \end{array}\right.\;(i,j=1,2,\ldots ,n)$$ (1)

Where, *b*_*ij*_ is the fuzzy correlation strength of factor *i* to factor *j*; *x*_*i*_, *x*_*j*_ is the sum of row *i* and column *j* of fuzzy incidence matrix *x*_*ij*_ respectively.

③ Select the threshold *λ*. The adjacency matrix *A* is determined by Eq (2).


$$a_{ij}=\left\{ \begin{array}{cc} 1 & b_{ij}>\lambda \\ 0 & b_{ij}\leq \lambda \end{array}\right.(i,j=1,2,\ldots ,n)\;Adjacency\;matrixA=(a_{ij}{)}_{n\times n}$$ (2)

④ Develop of reachability matrix. The matrix *A* should be developed further until it satisfifies the conditions of Eq (3) where the obtained matrix *M* is reachability matrix. This calculation process can be realized in Matlab.


$${\left(A+E\right)}^{1}\neq {\left(A+E\right)}^{2}\neq \ldots \neq {\left(A+E\right)}^{K-1}={\left(A+E\right)}^{K}=M$$ (3)

Where, *E* is an identity matrix.

⑤ Partition the factors into different levels. According to the reachability matrix *M* and Eqs (4)~(6), the antecedent set *A*(*S*_*j*_), the reachable set *R*(*S*_*i*_) and the intersection set *R*(*S*_*i*_)∩*A*(*S*_*j*_) are obtained. And the set *Q*(*S*_*i*_) of all the factors that can reach *S*_*i*_. The highest element is determined by Eq (6). Next, the first level factors will be determined and removed from the matrix. Repeating this method to determine the highest level feature set of each level, and dividing all factors into corresponding levels. Finally, the hierarchical structure digraph of the influencing factors of the hidden cost of prefabricated buildings is obtained.


$$R(S_{i})=\left\{S_{j}|S_{j}\in S,S_{\mathrm{ij}}=1\right\}$$ (4)

$$A(S_{j})=\left\{S_{i}|S_{i}\in S,S_{\mathrm{ij}}=1\right\}$$ (5)

$$Q(S_{i})=\{S_{i}|S_{i}\in S,R(S_{i})\cap A(S_{i})=R(S_{i})\}$$ (6)

### 3.3 Bayesian network

Bayesian network (BN) proposed by American professor Pearl. BN, also known as belief networks, causal networks or influence diagrams, are probabilistic network models. It is an uncertain probability graph reasoning model based on Bayesian theory [49, 50], and Bayes’s theorem is as follows (7).


$$P(X_{1}|X_{2})=\frac{P(X_{1})P(X_{2}|X_{1})}{P(X_{2})}$$ (7)

Where *P*(*X*_1_) and *P*(*X*_2_) are the prior probability of the parent node *X*_1_ and child node *X*_2_, and *P*(*X*_1_|*X*_2_) and *P*(*X*_2_|*X*_1_) are the prior and posterior conditional probability, respectively.

BN structure can be expressed as *B* = <*A*,*V*,*P*>, where, *V* = (*V*_1_,*V*_2_,…,*V*_*n*_) is the set of node variables; *n* represents the number of node variables; *A* = (*A*_12_,*A*_13_,…) is a directed edge set, which represents the causality or dependency between node variables; *P* is the local probability distribution set of the correlation between the child nodes and its parent nodes; Each node represents a random variable with two states of 0 (not occurrence) or 1 (occurrence). According to the chain rule and D-separation criterion, the joint probability of multiple variables *V* = (*V*_1_,*V*_2_,…,*V*_*n*_) can be given by the product of the conditional probabilities:

$$P(V)=P(V_{1},\ldots ,V_{n})=\prod_{i=1}^{n}P(V_{i}|Parents(V_{i}))$$ (8)

Where *P*(*V*) is the joint distribution of variables; *Parents*(*V*_*i*_) represents the collection of parent nodes of *V*_*i*_.

①Causal Reasoning: Given the node state, calculate the probability of the results in this state.

The conditional probability of the hidden cost is calculated, and it is divided into two parts: the conditional probability without evidence *V*_*i*_ based on prior knowledge and the conditional probability of evidence *V*_*t*_ based on the sample evidence in the construction process. When *U* node occurs (determined under the condition of *U* = 1), the probability *P*(*U* = 1|*V*_*i*_) is calculated as follows:

$$P(U=1|V_{i})=P(U=1|V_{1}=x_{1},V_{2}=x_{2},\ldots ,V_{n}=x_{n})=\frac{P(U=1,V_{1}=x_{1},V_{2}=x_{2},\ldots ,V_{n}=x_{n})}{P(V_{1}=x_{1},V_{2}=x_{2},\ldots ,V_{n}=x_{n})},\;V\in V_{t},V_{i}\in \{0,1\}$$ (9)

In this way, the probability of hidden cost of prefabricated buildings can be predicted. Therefore, the construction side of employer can take corresponding management measures in advance to reduce the probability of total cost expenditure.

② Diagnostic Reasoning: Predicting the cause probability of the result when the results are given

Under the condition that the hidden cost is known or not, the key factors causing the hidden cost is diagnosed by BN calculation, and the posterior probability is obtained. Assuming that the node *U* is the posterior probability distribution of each node in the occurrence state, the posterior probability of the occurrence of the *i* node *V*_*i*_ is *P*(*V*_*i*_ = 1|*U* = 1), which is calculated as follows:

$$P(V_{i}=1|U=1)=\frac{P(V_{i}=1)P(U=1|V_{i}=1)}{P(U=1)}\;i=1,2,3,\ldots ,n$$ (10)

## 4. Model application and result analysis

### 4.1 Modeling based on FISM-BN

In this paper, the parameters of the fuzzy part are fuzzy direct relation matrix *X*, fuzzy relation strength matrix *B*_*n*_ and adjacency matrix *A*. Firstly, the fuzzy direct relation matrix *X* is obtained by Delphi Expert scoring method, then the fuzzy relation strength matrix *B*_*n*_ is calculated, and then the adjacency matrix *A* is calculated by membership function. Delphi method is used to determine the correlation between 13 factors, and the scores are given according to 1.0, 0.9, 0.8, 0.7, 0.6, 0.5, 0.4, 0.3, 0.2, 0.1,0 (the greater the value is, the higher the correlation degree). According to the research experience and achievements of some scholars, the ideal expected effect can be achieved by 4~10 members of ISM Group [51]. Therefore, 15 experienced experts were invited, including 4 professors of relevant majors in Colleges and universities, 6 person (i.e. 2 project managers, 2 business managers, 1 construction worker and 1 estimator with more than 5 years of relevant working experience), 3 senior engineers and 2 government staffs. In order to avoid the subjective arbitrariness of expert evaluation, Delphi Expert scoring method, incentive principle, anonymous feedback and so on were used. Through the analysis of the factors *S*1~*S*14 (the hidden cost of prefabricated buildings), the relationship between them were analyzed. The fuzzy direct relation matrix *X* was obtained, as shown in Table 3.

**Table 3:** The fuzzy direct relation matrix *X*.

Si	S1	S2	S3	S4	S5	S6	S7	S8	S9	S10	S11	S12	S13	S14	Sum
S1	0	0.9	0.2	0.2	0.4	0.2	0.1	0.7	0.1	0.1	0.2	0.1	0.2	1	4.4
S2	0.1	0	0.2	0.1	0.3	0.1	0.2	0.1	0.1	0.2	0.2	0.1	0.2	0.9	2.8
S3	0.4	1	0.0	0.2	0.4	0.3	0.3	0.9	0.1	0.2	0.3	0.2	0.9	1	6.2
S4	0.1	0.2	0.1	0	0.8	0.7	0.3	0.2	0.6	0.3	0.2	0.3	0.1	1	4.9
S5	0.2	0.2	0.2	0.1	0	0.9	0.1	0.1	0.1	0.1	0.2	0.2	0.2	0.9	3.5
S6	0.1	0.1	0.1	0.1	0.1	0	0.2	0.2	0.2	0.2	0.1	0.1	0.3	0.8	2.6
S7	0.7	0.8	0.2	0.1	0.2	0.1	0	0.9	0.2	0.2	0.2	0.3	0.1	1	5
S8	0.3	0.8	0.1	0.2	0.1	0.3	0.2	0	0.2	0.2	0.1	0.1	0.3	1	3.9
S9	0.2	0.4	0.2	0.1	1	1	0.2	0.4	0	0.1	0.3	0.3	0.2	1	5.4
S10	0.3	0.4	0.7	0.3	0.9	0.1	0.1	0.4	0.2	0	0.2	0.2	0.9	1	5.7
S11	0.2	0.1	0.8	0.2	0.8	0.1	0.2	0.2	0.1	0.2	0	0.2	0.3	0.9	4.3
S12	0.2	0.3	0.2	0.1	0.1	0.2	0.2	0.3	0.2	0.1	0.2	0	1	0.9	4
S13	0.2	0.3	0.2	0.1	0.3	0.1	0.2	0.1	0.2	0.2	0.2	0.1	0	1	3.2
S14	0.1	0.1	0.2	0.1	0.2	0.1	0.1	0.2	0.1	0.1	0.1	0.1	0.2	0	1.7
Sum	3.1	5.6	3.4	1.9	5.6	4.2	2.4	4.7	2.4	2.2	2.5	2.3	4.9	12.4	

According to Eq (1), on the principle that the minority was subordinate to the majority, the fuzzy relation strength matrix *B*_*n*_ was finally obtained, as shown in Table 4.

**Table 4:** The fuzzy relation strength matrix *B*_*n*_.

Si	S1	S2	S3	S4	S5	S6	S7	S8	S9	S10	S11	S12	S13	S14
S1	0	0.10	0.03	0.03	0.04	0.02	0.01	0.08	0.01	0.02	0.03	0.02	0.02	0.06
S2	0.02	0	0.03	0.02	0.04	0.01	0.04	0.01	0.02	0.04	0.04	0.02	0.03	0.06
S3	0.04	0.09	0	0.03	0.04	0.03	0.04	0.09	0.01	0.02	0.04	0.02	0.09	0.06
S4	0.01	0.02	0.01	0	0.08	0.08	0.04	0.02	0.09	0.04	0.03	0.04	0.01	0.06
S5	0.03	0.02	0.03	0.02	0	0.13	0.02	0.01	0.02	0.02	0.03	0.04	0.02	0.06
S6	0.02	0.01	0.02	0.02	0.01	0	0.04	0.03	0.04	0.04	0.02	0.02	0.04	0.06
S7	0.09	0.08	0.02	0.01	0.02	0.01	0	0.10	0.03	0.03	0.03	0.04	0.01	0.06
S8	0.04	0.09	0.01	0.04	0.01	0.04	0.03	0	0.03	0.03	0.02	0.02	0.04	0.07
S9	0.02	0.04	0.02	0.01	0.10	0.12	0.03	0.04	0	0.01	0.04	0.04	0.02	0.06
S10	0.04	0.04	0.08	0.04	0.09	0.01	0.01	0.04	0.03	0	0.03	0.03	0.09	0.06
S11	0.03	0.01	0.12	0.03	0.09	0.01	0.03	0.02	0.02	0.03	0	0.03	0.03	0.06
S12	0.03	0.03	0.03	0.02	0.01	0.03	0.03	0.04	0.03	0.02	0.03	0	0.13	0.06
S13	0.03	0.04	0.03	0.02	0.04	0.01	0.04	0.01	0.04	0.04	0.04	0.02	0	0.07
S14	0.02	0.01	0.04	0.03	0.03	0.02	0.02	0.03	0.02	0.03	0.04	0.03	0.03	0

The smaller the threshold *λ* is, the rougher the hierarchical relationship of factors are. If the hierarchical relationship of factors are too fine or too rough, it won’t reflect the relationship between factors, so *λ* should be appropriate. According to the experimental method, *λ* = 0.04 was selected, and the adjacency matrix *A* was established by Eq (2), as shown in Table 5.

**Table 5:** The adjacency matrix *A*.

Si	S1	S2	S3	S4	S5	S6	S7	S8	S9	S10	S11	S12	S13	S14
S1	0	1	0	0	0	0	0	1	0	0	0	0	0	1
S2	0	0	0	0	0	0	0	0	0	0	0	0	0	1
S3	0	1	0	0	0	0	0	1	0	0	0	0	1	1
S4	0	0	0	0	1	1	0	0	1	0	0	0	0	1
S5	0	0	0	0	0	1	0	0	0	0	0	0	0	1
S6	0	0	0	0	0	0	0	0	0	0	0	0	0	1
S7	1	1	0	0	0	0	0	1	0	0	0	0	0	1
S8	0	1	0	0	0	0	0	0	0	0	0	0	0	1
S9	0	0	0	0	1	1	0	0	0	0	0	0	0	1
S10	0	0	1	0	1	0	0	0	0	0	0	0	1	1
S11	0	0	1	0	1	0	0	0	0	0	0	0	0	1
S12	0	0	0	0	0	0	0	0	0	0	0	0	1	1
S13	0	0	0	0	0	0	0	0	0	0	0	0	0	1
S14	0	0	0	0	0	0	0	0	0	0	0	0	0	0

**Fig 3:**
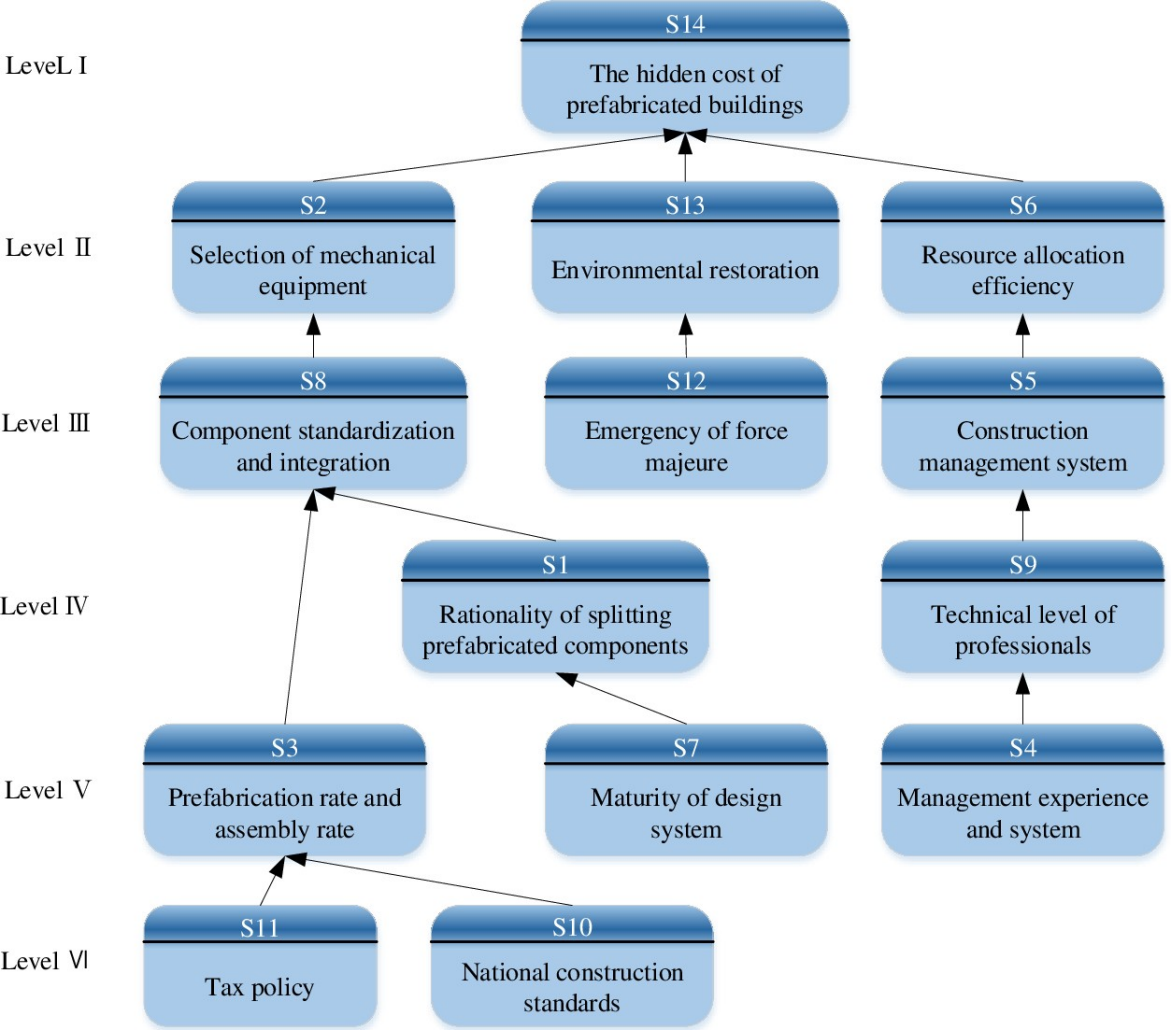
Hierarchical structure digraph.

On the basis of adjacency matrix *A*, the reachability matrix*M* was calculated by Eq (3) in Matlab 2018a, as shown in Table 6. By using Eqs (4)~(6), the antecedent set and reachable set were obtained, and the level decomposition layer was shown in Table 7. Finally, the hierarchical structure of the influencing factors were obtained, as shown in Fig 3.

**Table 6:** The reachability matrix *M*.

Si	S1	S2	S3	S4	S5	S6	S7	S8	S9	S10	S11	S12	S13	S14
S1	1	1	0	0	0	0	0	1	0	0	0	0	0	1
S2	0	1	0	0	0	0	0	0	0	0	0	0	0	1
S3	0	1	1	0	0	0	0	1	1	0	0	0	1	1
S4	0	0	0	1	1	1	0	0	1	0	0	0	0	1
S5	0	0	0	0	1	1	0	0	0	0	0	0	0	1
S6	0	0	0	0	0	1	0	0	0	0	0	0	0	1
S7	1	1	0	0	0	0	1	1	0	0	0	0	0	1
S8	0	1	0	0	0	0	0	1	0	0	0	0	0	1
S9	0	0	0	0	1	1	0	0	1	0	0	0	0	1
S10	0	1	1	0	1	1	0	1	0	1	0	0	1	1
S11	0	1	1	0	1	1	0	1	0	0	1	0	1	1
S12	0	0	0	0	0	0	0	0	0	0	0	1	1	1
S13	0	0	0	0	0	0	0	0	0	0	0	0	1	1
S14	0	0	0	0	0	0	0	0	0	0	0	0	0	1

**Table 7:** Reachable set, antecedent set and common set.

Factor	*R*(*S*_*i*_)	*A*(*S*_*j*_)	*Q*(*S*_*i*_)	Level
S1	1.2.8.14	1.7	1	4
S2	2.14	1~3.7.8.10.11	2	2
S3	2.3.8.9.13.14	3.10.11	3	5
S4	4~6.9.14	4	4	5
S5	5.6.14	4.5.9~11	5	3
S6	6.14	4~6.9~11	6	2
S7	1.2.7.8.14	7	7	5
S8	2.8.14	1.3.7.8.10.11	8	3
S9	5.6.9.14	4.9	9	4
S10	2.3.5.6.8.10.13.14	10	10	6
S11	2.3.5.6.8.11.13.14	11	11	6
S12	12~14	12	12	3
S13	13.14	3.10~13	13	2
S14	14	1~14	14	1

**Fig 4:**
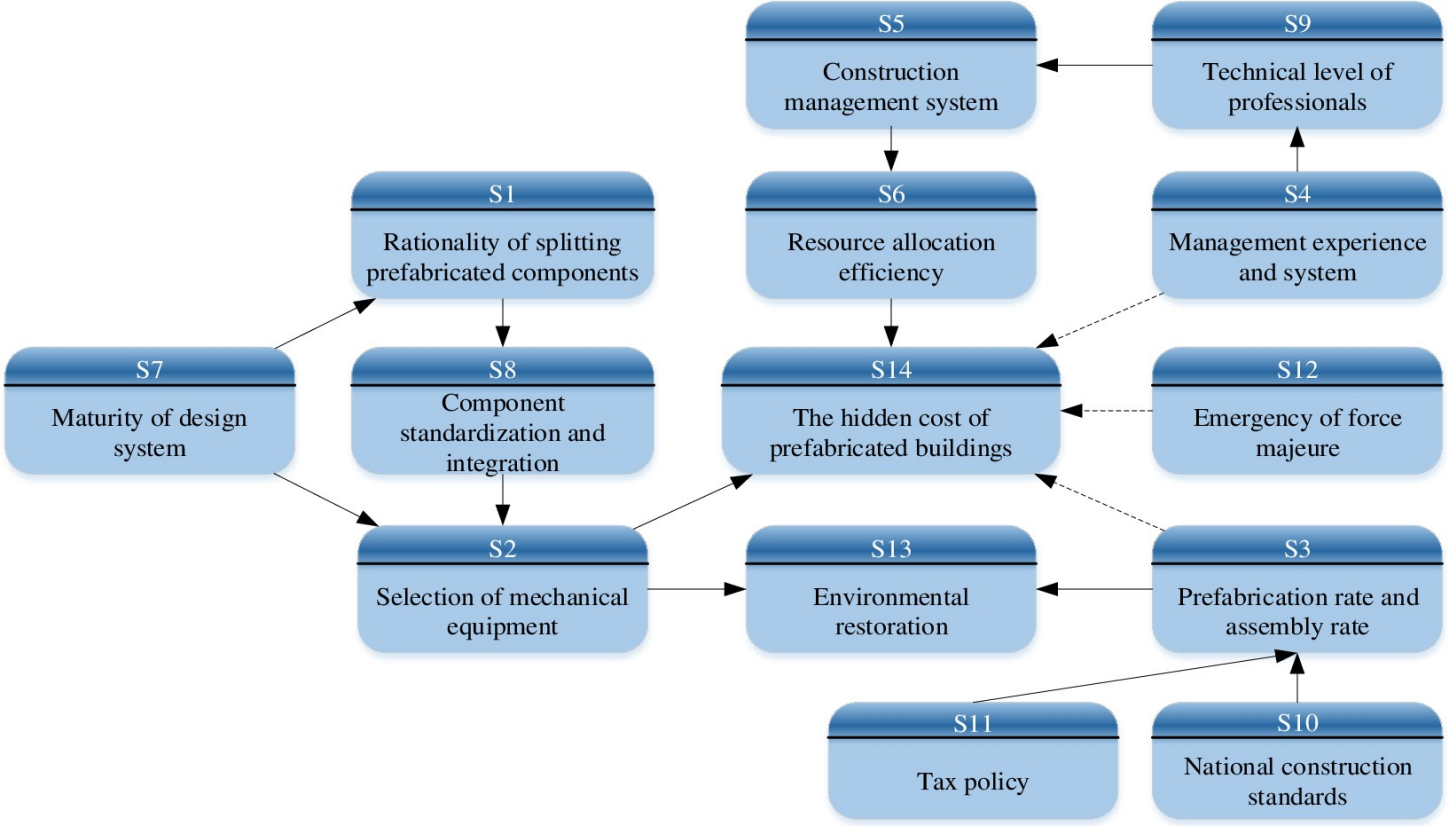
Bayesian network of hidden cost of prefabricated buildings.

As FISM ignores the skip-level relationship and there is no feedback loop between stages. It will lead to some important correlation between the influencing factors that can not be reflected. Therefore, on the basis of the hierarchical structure model, referring to the causality diagram method in reference [52]. The BN model was modified, as shown in Fig 4. The dotted arrow is a new added relationship. The revision principles of causality diagram method are as follows:

STEP1: Distinguishing between direct and indirect relationships. The factor direct and indirect relationship distinguishing. Although prefabrication rate and assembly rate (S3) could indirectly affect the hidden cost of prefabricated buildings (S14) through environmental restoration (S13), there was a direct internal relationship. It should add a relationship adjacency arrow between them. Similarly, management experience and system (S4), Emergency of force majeure (S12) and the hidden cost of prefabricated buildings(S14) had a direct impact. There was also a direct impact between the maturity of design system (S7) and the selection of mechanical equipment (S2). Therefore, it should add a connection line between them respectively.
STEP2: Conditional independencies. Conditional independence has been achieved in Fig 4.
STEP3: Checking factors causal inversion. There has been no causal inversion in Fig 4.
STEP4: Eliminating circular relations. There has been no circular causality in Fig 4.

### 4.2 Parameter determination of Bayesian network model

Through the analysis of literature and experts, the interview results had been analyzed and corrected repeatedly. The prior probability of each root nodes in Fig 4 and the connection probability of other influencing factors with the parent nodes were finally determined, as shown in Table 8. Then, the connection probability between the child nodes, the parent nodes and the conditional probability table (CPT) of each node was calculated by using the Leak noisy or gate model [53] and Eqs (7) and (8). As shown in Fig 4, the connection probabilities of sub node S3 with its parent node S10, S11 and other influencing factors were 53.3%, 40.2% and 15%, respectively. The CPT of sub node S3 was shown in Table 9. The calculation process of other sub nodes were the same as that of S3, and it would not be listed one by one due to the length of an article. The CPT of the remaining nodes were shown in the (S7 File).

**Table 8:** Prior probability of root nodes.

Factors	S4		S7		S10		S11		S12	
Event Status	Y	N	Y	N	Y	N	Y	N	Y	N
Prior Probability	6%	94%	6%	94%	3%	97%	3%	97%	1%	99%

**Table 9:** CPT of sub node S3.

Parent Node Status					
Factors	S10	Y		N	
	S11	Y	N	Y	N
Conditional Probability of S3	Y	76.3%	60.3%	49.2%	15%
	N	23.7%	39.7%	50.8%	85%

### 4.3 Causal reasoning analysis

**Fig 5:**
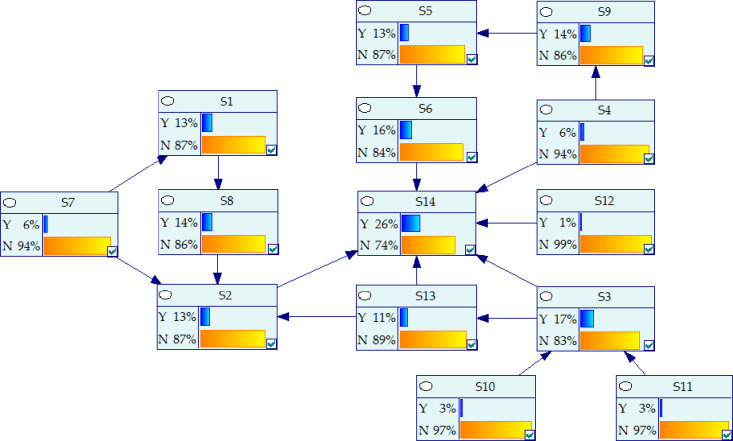
Causal reasoning results.

**Fig 6:**
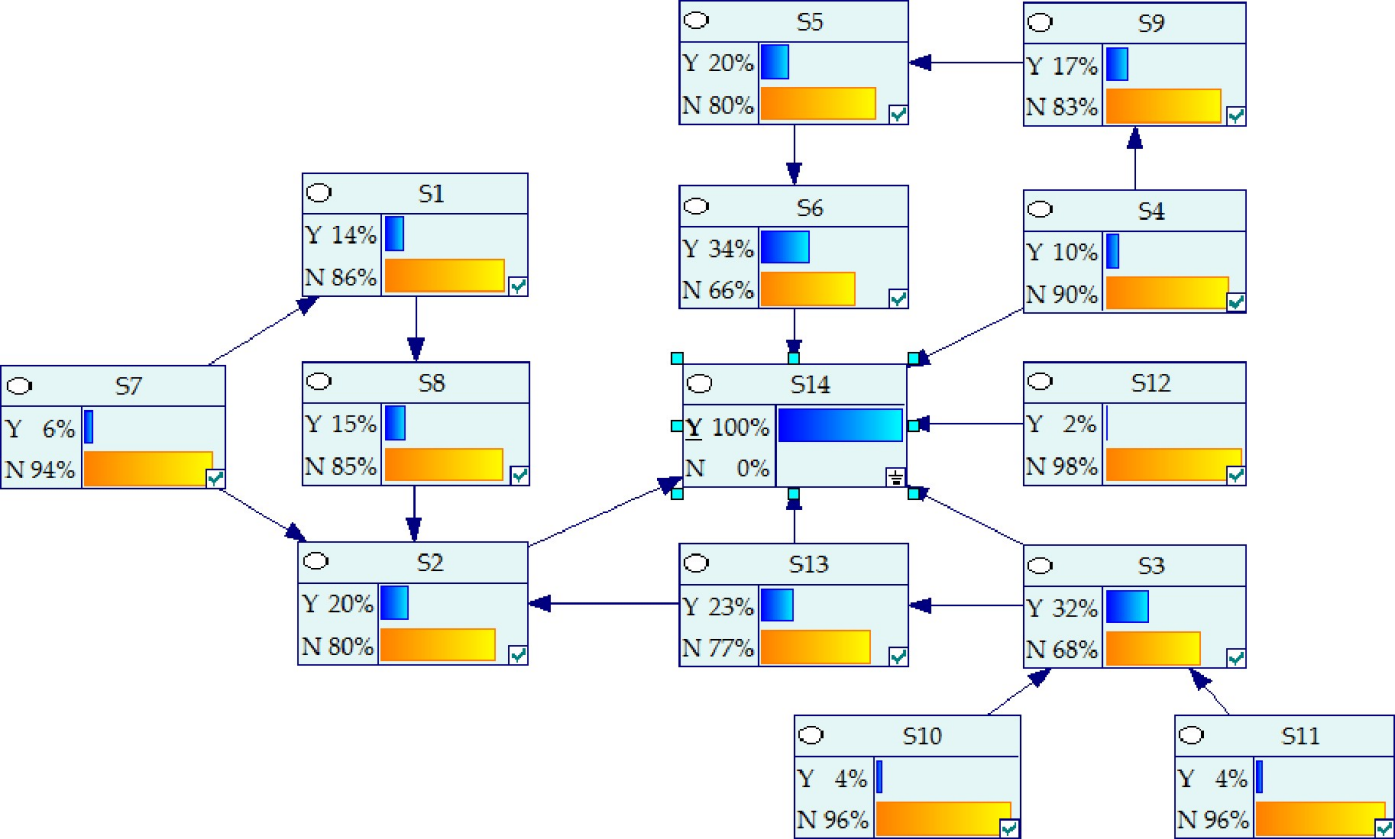
Diagnostic reasoning results.

After the construction of BN model, the relationship among the factors in Fig 4 were input to GeNIe2.0. Then the CPT of all nodes were imported, and the prior probability of each node was calculated in GeNIe2.0 using Eq (9). The prior probability was 26%, as shown in Fig 5. It could be concluded that the prior probability of the hidden cost was small. In Figs 5 and 6, Y indicates occurrence and N indicates not occurrence. When the evidence conditions were introduced, the evidence conditions would pass layer by layer through the BN model of the hidden cost, and the probability of hidden cost under different conditions could be calculated. As there were too many nodes in this paper, only the state of root nodes was assumed to calculate the probability of hidden cost, as shown in Table 10. And the relevant data was included in the S8 File.

**Table 10:** Probability of hidden cost of prefabricated buildings.

Probability Type	Evidence	Probability
Prior Probability	\	26%
Posterior Probability	*P*(*S*4 = 1) = 1	47%
	*P*(*S*7 = 1) = 1	28%
	*P*(*S*10 = 1) = 1	38%
	*P*(*S*11 = 1) = 1	35%
	*P*(*S*12 = 1) = 1	44%
	*P*(*S*4 = 0) = 1	25%
	*P*(*S*12 = 1) = 1	57%
	*P*(*S*4 = 1) = 1	
	*P*(*S*11 = 1) = 1	

It could be seen from Table 10 that when a root node was known, the probability of hidden cost occurrence was increased compared with its prior probability. When a root node was known not to happen, the probability of hidden cost occurrence was reduced compared with its prior probability. When several root nodes were known to occur at the same time, such as S4, S11 and S12, the probability of hidden cost increased from 26% to 57%. It could be concluded that the probability of multiple nodes occurring at the same time was higher than that of single node. This was also in line with the actual situation. Therefore, it could be concluded that there was a positive correlation between the influencing factors in BN model and the hidden cost of prefabricated buildings. This also indirectly proved that the BN model of the hidden cost was feasible.

### 4.4 Diagnostic reasoning analysis

BN was used to diagnose the hidden cost of prefabricated buildings. According to Eq (10), the influence degree of each node on hidden cost was calculated and deduced in GeNIe2.0. As shown in Fig 6, assuming that the hidden cost of prefabricated buildings (S14) occurred, the probability value of each node led to *P*(*S*_14_ = 1) = 1 that could be inversely deduced. And the probability value of each node was sorted. The larger the probability of nodes occurrence, the greater the impact on the hidden cost, thus the key factors leading to the occurrence of hidden cost could be obtained. It could also indirectly find out the reasons for the increase of total project cost. In this way, targeted measures could be taken to optimize the hidden cost, so as to achieve reasonable control of the total project cost.

When the hidden cost of prefabricated buildings occurs, the probability of each influencing factor was shown in Table 11. Resource allocation efficiency (S6), prefabrication rate and assembly rate (S3) and environmental restoration (S13) had high probability, which were 34%, 32% and 23% respectively. This showed that the three factors most likely led to the occurrence of hidden cost. Therefore, after the hidden cost occurred, S3, S6, S13 should be given priority. And the possibility of influencing factors was investigated one by one. When new evidence was found in the investigation, such as *P*(*S*_2_ = 0) = 1 and *P*(*S*_11_ = 0) = 1, the probability of other nodes could be calculated by inputting it into GeNIe2.0. By constantly inputting new evidence, the probability of other nodes were constantly updated, and corresponding control measures were taken for the factors with high probability of node occurrence until the hidden cost was controlled. The probability of occurrence of national construction standard (S10), tax policy (S11) and Emergency of force majeure (S12) were small, they were 4%, 4% and 2% respectively. However, when the hidden cost occurs, it is easy to judge the occurrence state of the three according to the actual situation, so it should also be taken as the priority analysis object.

**Table 11:** Ranking of occurrence probability of influencing factors.

Factors	Probability	Ranking	Factors	Probability	Ranking
S6	34%	1	S1	14%	7
S3	32%	2	S4	10%	8
S13	23%	3	S7	6%	9
S2	20%	4	S10	4%	10
S5	20%	4	S11	4%	10
S9	17%	5	S12	2%	11
S8	15%	6			

## 5. Conclusion

In this paper, 13 key factors influencing the hidden cost were determined from five dimensions i.e. design, management, technology, policy and environment. The importance and characteristics of hidden cost of prefabricated buildings were analyzed. The large quantities of hidden cost data are not easy to be obtained and the data has great uncertainty. There is also a complex correlation between the influencing factors of the hidden cost, and one single model is difficult to overcome these problems at the same time. BN has no requirement on the size of sample data, and it has a relatively mature application in the uncertainty problem. Meanwhile, FISM can visually represent the fuzzy and complex relationship between the influencing factors of hidden cost with graphics. Therefore, the FISM-BN model was used to analyze the hidden cost of prefabricated buildings.

And the probability of hidden cost occurrence of was 26% through causal reasoning. When new evidence was input, the probability of hidden cost could be updated continuously. The reverse diagnosis reasoning could find out the key factors that affect the hidden cost of prefabricated buildings when they occurred, and then checked and determined the final influencing factors one by one. Then targeted measures could be taken to reduce project costs. The model not only can deal with the data with large uncertainty, but also has no requirement for the size of sample data, and can directly reflect the complex internal relationship of factors. Dynamic analysis of hidden cost according to the change of actual project promotion by this model. However, if there are too many influencing factors, FISM will be cumbersome to use, and when there are many and complex nodes in BN, it is difficult to calculate the CPT of each node. In addition, the influencing factors system of hidden cost of prefabricated buildings obtained by literature analysis and questionnaire has certain subjectivity. These problems should be taken into account and solved in the future research.

The research innovatively studies the prefabricated buildings cost from the perspective of hidden cost, establishes the index system of the hidden cost, and constructs the FISM-BN analysis mode. This provides a reference for managers to manage the prefabricated buildings cost in the actual project, and also provides a new idea for the research, management and theoretical basis of prefabricated buildings cost.

## Supporting information

S1 File Questionnaire file. (PDF)

S2 File Questionnaire data file. (XLSX)

S3 File Consent letter file. (DOCX)

S4 File Interview file. (DOCX)

S5 File Expert information file. (XLSX)

S6 File Data of T-value test and variance test file. (DOCX)

S7 File Data of CPT of sub node file. (DOCX)

S8 File Data of the points extracted from images for analysis. (ZIP)
