# Supplementary material for: Analysis on the hidden cost of prefabricated buildings based on FISM-BN
Source: PLoS One. 2021 Jun 3;16(6):e0252138. doi: 10.1371/journal.pone.0252138 (PMC8174746; doi:10.1371/journal.pone.0252138)
Supplement: S1 File — (PDF) [file pone.0252138.s001.pdf]

Questionnaire Survey on Influencing Factors of Hidden Cost of —— Prefabricated Buildings

Thank you very much for taking time out of your busy schedule to fill in this questionnaire! The purpose of this survey is to provide valuable data for "determining the influencing factors of the hidden cost of prefabricated buildings".

I hope you can refer to the relevant information of prefabricated buildings cost analysis and your own work experience to answer truthfully. **All data results of this questionnaire will only be used for academic research. If you agree to authorize, please fill in this questionnaire.**

Thank you for your help and support! I wish you every success and good health!

Section 1: Basic information survey

1.Understanding of prefabricated buildings

- ○ Very understanding
- ○ Relatively understanding
- ○ General understanding
- ○ Not very understanding

2.Understanding of hidden cost of construction project

- ○ Very understanding
- ○ Relatively understanding
- ○ General understanding
- ○ Not very understanding

3. Nature of work unit

- ◯ Owner
- ◯ Contractor
- ◯ Designer
- ◯ Supervision
- ◯ Institutions of higher learning
- ◯ Manufacturers of prefabricated components

4.Working years engaged in prefabricated buildings

- ◯ 1-2years
- ◯ 3-5years
- ◯ 6-7years
- ◯ More than 7 years

5.Educational background

- ◯ Below bachelor degree
- ◯ Bachelor degree
- ◯ Master degree
- ◯ Doctor degree or above

6.Professional ranks and titles

- ◯ Primary title
- ◯ Middle title
- ◯ Vice-senior title
- ◯ Senior title

Section 2: Investigation on the importance of influencing factors of hidden cost of prefabricated buildings

This section is mainly to investigate the importance and rationality of 16 indicators. Please refer to the relevant information of prefabricated buildings cost analysis and your own work experience to evaluate and score the influencing factors, and tick "√" in the following scoring table. (The scoring rules are as follows: 1-Unimportant; 2-Generally important; 3-Relatively important; 4-Important; 5-Very important.)

7.Design Factors

	UI	GI	I	RI	VI
**Rationality of splitting prefabricated components**					
**Selection of mechanical equipment**					
**Prefabrication rate and assembly rate**					
**the site selection of prefabrication plant**					

8.Management Factors

	UI	GI	RI	I	VI
**Management experience and system**					
**Construction management system**					
**Resource allocation efficiency**					
**Integrity of industrial chain**					

9.Technology Factors

	UI	GI	RI	I	VI
**Component standardization and integration**					
**Technical level of professionals**					

10.Policy Factors

	UI	GI	RI	I	VI
**National construction standards**					
**Tax policy**					
**Engineering construction standard**					

11.Environment Factors

	UI	GI	RI	I	VI
**Emergency of force majeure**	◯	◯	◯	◯	◯
**Environmental restoration**	◯	◯	◯	◯	◯

Section 3: Opinions and suggestions

Your opinion on this questionnaire survey:____

____

Your suggestion on this questionnaire survey:______________________________________

____
