# Supplementary material for: Analysis on the hidden cost of prefabricated buildings based on FISM-BN
Source: PLoS One. 2021 Jun 3;16(6):e0252138. doi: 10.1371/journal.pone.0252138 (PMC8174746; doi:10.1371/journal.pone.0252138)
Supplement: S3 File — (DOCX) [file pone.0252138.s003.docx]

**Interview Consent**

Dear Sir/Madam:

With the increasing support of environmental protection and resource conservation in China, prefabricated buildings have been greatly supported and promoted in various places. But the high cost has been one of the main reasons restricting the development of prefabricated buildings.

From the perspective of hidden cost, this research group hopes to analyze the relationship between the influencing factors of the hidden cost of prefabricated buildings, and explore how to manage the hidden cost well.

We guarantee that the interview results and all relevant information obtained in the interview will only be used in academic research. The relevant information of the interviewee is strictly confidential.

Thank you very much for your support!

Research Group on prefabricated buildings cost
