# Supplementary material for: Analysis on the hidden cost of prefabricated buildings based on FISM-BN
Source: PLoS One. 2021 Jun 3;16(6):e0252138. doi: 10.1371/journal.pone.0252138 (PMC8174746; doi:10.1371/journal.pone.0252138)
Supplement: S4 File — (DOCX) [file pone.0252138.s004.docx]

Dear Expert:

In order to study the relationship between the influencing factors of the hidden cost of prefabricated buildings. This interview gives 13 influencing factors on the hidden cost of prefabricated buildings, as shown in Table 1. Please score in Table 2 according to 1.0, 0.9, 0.8, 0.7, 0.6, 0.5, 0.4, 0.3, 0.2, 0.1, 0. The greater the score, the greater the influence relationship.

**Table 1. Influencing factors of hidden cost of prefabricated buildings**

| **Target layer** | **Factors** | **Factors** |
| --- | --- | --- |
| The  Hidden  Cost  of prefabricated buildings  (S14) | Rationality of splitting prefabricated components (S1) | Component standardization and integration (S8) |
|  | Selection of mechanical equipment (S2) | Technical level of professionals (S9) |
|  | Prefabrication rate and assembly rate (S3) | National construction standards (S10) |
|  | Management experience and system (S4) | Tax policy (S11) |
|  | Construction management system (S5) | Emergency of force majeure (S12) |
|  | Resource allocation efficiency (S6) | Environmental restoration (S13) |
|  | Maturity of design system (S7) |  |

**Table 2. Scoring table of factor correlation**

|  | **S1** | **S2** | **S3** | **S4** | **S5** | **S6** | **S7** | **S8** | **S9** | **S10** | **S11** | **S12** | **S13** | **S14** |
| --- | --- | --- | --- | --- | --- | --- | --- | --- | --- | --- | --- | --- | --- | --- |
| S1 | 0 |  |  |  |  |  |  |  |  |  |  |  |  |  |
| S2 |  | 0 |  |  |  |  |  |  |  |  |  |  |  |  |
| S3 |  |  | 0 |  |  |  |  |  |  |  |  |  |  |  |
| S4 |  |  |  | 0 |  |  |  |  |  |  |  |  |  |  |
| S5 |  |  |  |  | 0 |  |  |  |  |  |  |  |  |  |
| S6 |  |  |  |  |  | 0 |  |  |  |  |  |  |  |  |
| S7 |  |  |  |  |  |  | 0 |  |  |  |  |  |  |  |
| S8 |  |  |  |  |  |  |  | 0 |  |  |  |  |  |  |
| S9 |  |  |  |  |  |  |  |  | 0 |  |  |  |  |  |
| S10 |  |  |  |  |  |  |  |  |  | 0 |  |  |  |  |
| S11 |  |  |  |  |  |  |  |  |  |  | 0 |  |  |  |
| S12 |  |  |  |  |  |  |  |  |  |  |  | 0 |  |  |
| S13 |  |  |  |  |  |  |  |  |  |  |  |  | 0 |  |
| S14 |  |  |  |  |  |  |  |  |  |  |  |  |  | 0 |

Thank you for taking the time to participate in this interview!

Research Group on prefabricated buildings cost
