# Supplementary material for: Analysis on the hidden cost of prefabricated buildings based on FISM-BN
Source: PLoS One. 2021 Jun 3;16(6):e0252138. doi: 10.1371/journal.pone.0252138 (PMC8174746; doi:10.1371/journal.pone.0252138)
Supplement: S6 File — (DOCX) [file pone.0252138.s006.docx]

Table 1 T-value test

| Factors |  |  |  |  | 95% confidence interval of difference | | |
| --- | --- | --- | --- | --- | --- | --- | --- |
|  | T-value | 自由度 | Sig.（2-tailed） | Mean difference | | Upper bounds | Lower bounds |
| Rationality of splitting prefabricated components | 14.201 | 60 | .000 | 1.426 | | 1.23 | 1.63 |
| Selection of mechanical equipment | 18.448 | 60 | .000 | 1.623 | | 1.45 | 1.80 |
| Prefabrication rate and assembly rate | 15.331 | 60 | .000 | 1.459 | | 1.27 | 1.65 |
| The site selection of prefabrication plant | -2.650 | 60 | .000 | -.344 | | -.60 | -.08 |
| Management experience and system | 24.368 | 60 | .000 | 1.721 | | 1.58 | 1.86 |
| Construction management system | 28.658 | 60 | .000 | 1.787 | | 1.66 | 1.91 |
| Resource allocation efficiency | 12.400 | 60 | .000 | 1.361 | | 1.14 | 1.58 |
| The integrity of industrial chain | -1.447 | 60 | .000 | -.197 | | -.47 | .08 |
| Maturity of design system | 27.586 | 60 | .000 | 1.803 | | 1.67 | 1.93 |
| Component standardization and integration | 16.318 | 60 | .000 | 1.590 | | 1.40 | 1.79 |
| Technical level of professionals | 14.482 | 60 | .000 | 1.459 | | 1.26 | 1.66 |
| National construction standards | 10.701 | 60 | .000 | 1.279 | | 1.04 | 1.52 |
| Tax policy | 9.068 | 60 | .000 | 1.131 | | .88 | 1.38 |
| Engineering construction standard | 8.439 | 60 | .000 | 1.148 | | .88 | 1.42 |
| Emergency of force majeure | 6.621 | 60 | .000 | .836 | | .58 | 1.09 |
| Environmental restoration | -9.923 | 60 | .000 | 1.721 | | 1.56 | 1.88 |

Table 2 Variance test

| Factors | Number of samples | Mean | Standard deviations | Standard error of mean |
| --- | --- | --- | --- | --- |
| Rationality of splitting prefabricated components | 61 | 4.43 | .784 | .100 |
| Selection of mechanical equipment | 61 | 4.62 | .687 | .088 |
| Prefabrication rate and assembly rate | 61 | 4.46 | .743 | .095 |
| The site selection of prefabrication plant | 61 | 2.66 | 1.015 | .130 |
| Management experience and system | 61 | 4.72 | .552 | .071 |
| Construction management system | 61 | 4.79 | .487 | .062 |
| Resource allocation efficiency | 61 | 4.36 | .857 | .110 |
| The integrity of industrial chain | 61 | 2.80 | 1.062 | .136 |
| Maturity of design system | 61 | 4.80 | .511 | .065 |
| Component standardization and integration | 61 | 4.59 | .761 | .097 |
| Technical level of professionals | 61 | 4.46 | .787 | .101 |
| National construction standards | 61 | 4.28 | .933 | .119 |
| Tax policy | 61 | 4.13 | .974 | .125 |
| Engineering construction standard | 61 | 4.15 | 1.062 | .136 |
| Emergency of force majeure | 61 | 3.84 | .986 | .126 |
| Environmental restoration | 61 | 4.72 | .636 | .081 |
