# Supplementary material for: Analysis on the hidden cost of prefabricated buildings based on FISM-BN
Source: PLoS One. 2021 Jun 3;16(6):e0252138. doi: 10.1371/journal.pone.0252138 (PMC8174746; doi:10.1371/journal.pone.0252138)
Supplement: S7 File — (DOCX) [file pone.0252138.s007.docx]

**Table 1. CPT of sub node S1**

| **Parent Node Status** | | | |
| --- | --- | --- | --- |
| Factors | S7 | Y | N |
| Conditional Probability of S1 | Y | 0.636 | 0.100 |
|  | N | 0.364 | 0.900 |

**Table2. CPT of sub node S2**

| **Parent Node Status** | | | | | | | | | |
| --- | --- | --- | --- | --- | --- | --- | --- | --- | --- |
| Factors | S13 | Y | | | | N | | | |
|  | S8 | Y | | N | | Y | | N | |
|  | S7 | Y | N | Y | N | Y | N | Y | N |
| Conditional Probability of S2 | Y | 0.596 | 0.504 | 0.431 | 0.248 | 0.379 | 0.291 | 0.186 | 0.070 |
|  | N | 0.404 | 0.496 | 0.569 | 0.752 | 0.621 | 0.709 | 0.814 | 0.930 |

**Table 3. CPT of sub node S5**

| **Parent Node Status** | | | |
| --- | --- | --- | --- |
| Factors | S9 | Y | N |
| Conditional Probability of S5 | Y | 0.289 | 0.100 |
|  | N | 0.711 | 0.900 |

**Table 4. CPT of sub node S6**

| **Parent Node Status** | | | |
| --- | --- | --- | --- |
| Factors | S5 | Y | N |
| Conditional Probability of S6 | Y | 0.589 | 0.100 |
|  | N | 0.411 | 0.900 |

**Table 5. CPT of sub node S8**

| **Parent Node Status** | | | |
| --- | --- | --- | --- |
| Factors | S1 | Y | N |
| Conditional Probability of S8 | Y | 0.267 | 0.120 |
|  | N | 0.733 | 0.880 |

**Table 6. CPT of sub node S9**

| **Parent Node Status** | | | |
| --- | --- | --- | --- |
| Factors | S4 | Y | N |
| Conditional Probability of S9 | Y | 0.478 | 0.120 |
|  | N | 0.522 | 0.880 |

**Table 7. CPT of sub node S13**

| **Parent Node Status** | | | |
| --- | --- | --- | --- |
| Factors | S3 | Y | N |
| Conditional Probability of S13 | Y | 0.175 | 0.100 |
|  | N | 0.825 | 0.900 |

**Table 8. CPT of sub node S14**

| **Parent Node Status** | | | | | | | | | | | | | | | | | | | | | | | | | | | | | | | | | |
| --- | --- | --- | --- | --- | --- | --- | --- | --- | --- | --- | --- | --- | --- | --- | --- | --- | --- | --- | --- | --- | --- | --- | --- | --- | --- | --- | --- | --- | --- | --- | --- | --- | --- |
| Factors | S6 | Y | | | | | | | | | | | | | | | | | | | | | | | | | | | | | | | |
|  | S13 | Y | | | | | | | | | | | | | | | | N | | | | | | | | | | | | | | | |
|  | S2 | Y | | | | | | | | N | | | | | | | | Y | | | | | | | | N | | | | | | | |
|  | S3 | Y | | | | N | | | | Y | | | | N | | | | Y | | | | N | | | | Y | | | | N | | | |
|  | S12 | Y | | N | | Y | | N | | Y | | N | | Y | | N | | Y | | N | | Y | | N | | Y | | N | | Y | | N | |
|  | S4 | Y | N | Y | N | Y | N | Y | N | Y | N | Y | N | Y | N | Y | N | Y | N | Y | N | Y | N | Y | N | Y | N | Y | N | Y | N | Y | N |
| Conditional Probability of S14 | Y | .797 | .786 | .717 | .686 | .679 | .624 | .529 | .476 | .730 | .700 | .604 | .560 | .550 | .500 | .340 | .228 | .766 | .740 | .656 | .618 | .609 | .566 | .427 | .363 | .672 | .663 | .519 | .465 | .467 | .392 | .198 | .649 |
|  | N | .203 | .214 | .283 | .314 | .321 | .376 | .471 | .524 | .270 | .300 | .396 | .440 | .450 | .500 | .660 | .772 | .234 | .260 | .344 | .382 | .391 | .434 | .573 | .637 | .328 | .337 | .481 | .535 | .533 | .608 | .802 | .351 |
|  | | | | | | | | | | | | | | | | | | | | | | | | | | | | | | | | | |
| Parent Node Status | | | | | | | | | | | | | | | | | | | | | | | | | | | | | | | | | |
| Factors | S6 | N | | | | | | | | | | | | | | | | | | | | | | | | | | | | | | | |
|  | S13 | Y | | | | | | | | | | | | | | | | N | | | | | | | | | | | | | | | |
|  | S2 | Y | | | | | | | | N | | | | | | | | Y | | | | | | | | N | | | | | | | |
|  | S3 | Y | | | | N | | | | Y | | | | N | | | | Y | | | | N | | | | Y | | | | N | | | |
|  | S12 | Y | | N | | Y | | N | | Y | | N | | Y | | N | | Y | | N | | Y | | N | | Y | | N | | Y | | N | |
|  | S4 | Y | N | Y | N | Y | N | Y | N | Y | N | Y | N | Y | N | Y | N | Y | N | Y | N | Y | N | Y | N | Y | N | Y | N | Y | N | Y | N |
| Conditional Probability of S14 | Y | .794 | .772 | .699 | .665 | .657 | .619 | .498 | .442 | .712 | .680 | .578 | .531 | .52 | .467 | .297 | .633 | .750 | .722 | .634 | .593 | .584 | .537 | .389 | .321 | .650 | .611 | .487 | .430 | .417 | .352 | .537 | .050 |
|  | N | .206 | .228 | .301 | .335 | .343 | .381 | .502 | .558 | .288 | .320 | .422 | .469 | .480 | .533 | .703 | .367 | 0.25 | .278 | .366 | .407 | .416 | .463 | .611 | .679 | 0.35 | .389 | .513 | .570 | .583 | .648 | .463 | .950 |
