# Supplementary figures and images for: Analysis on the hidden cost of prefabricated buildings based on FISM-BN
Source: PLoS One. 2021 Jun 3;16(6):e0252138. doi: 10.1371/journal.pone.0252138 (PMC8174746; doi:10.1371/journal.pone.0252138)

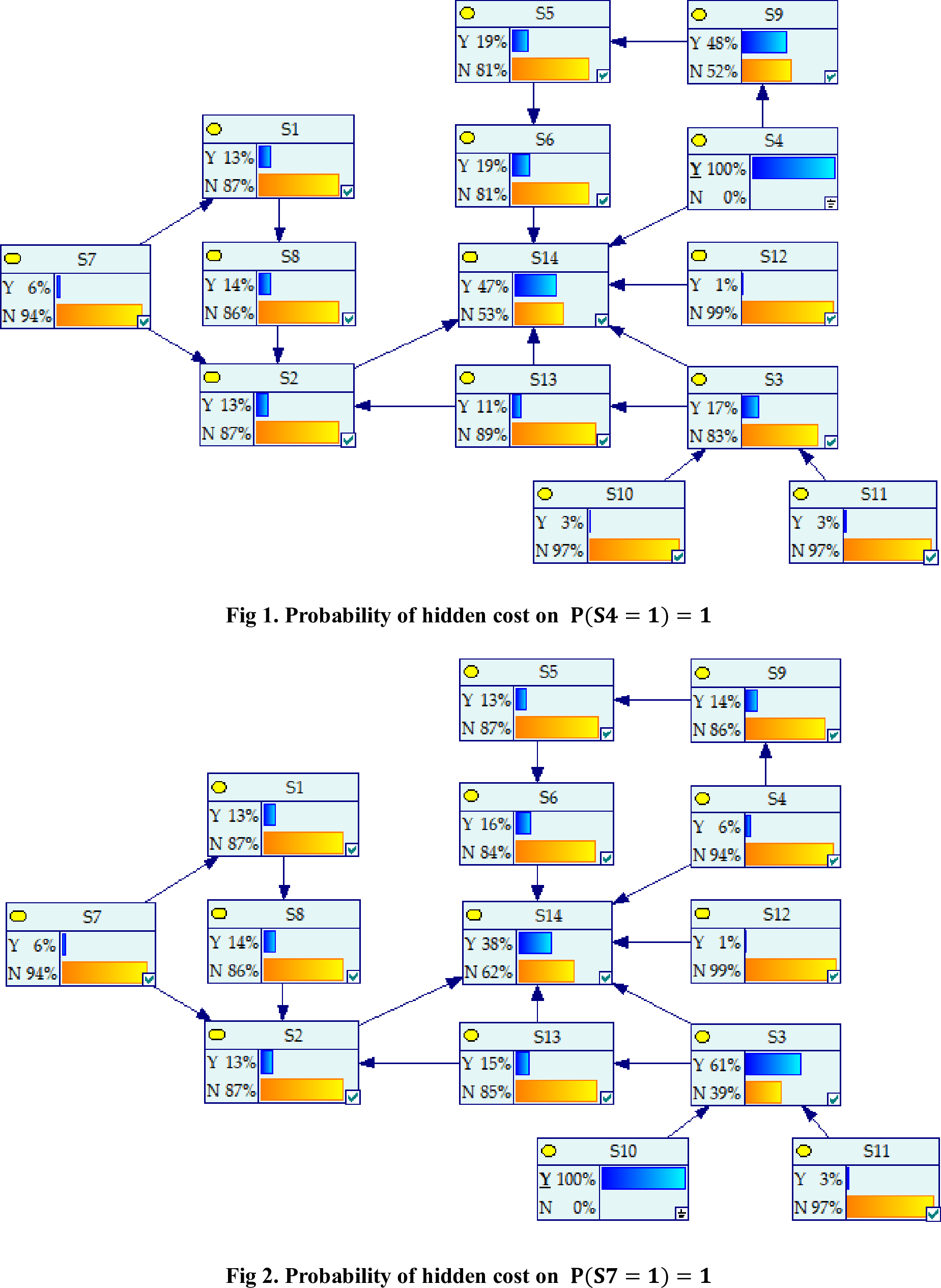

Supplement: S8 File — (ZIP) [file pone.0252138.s008.zip › S8 Data of the points extracted from images for analysis/S8 1.Data of the points extracted from images for analysis.tif]

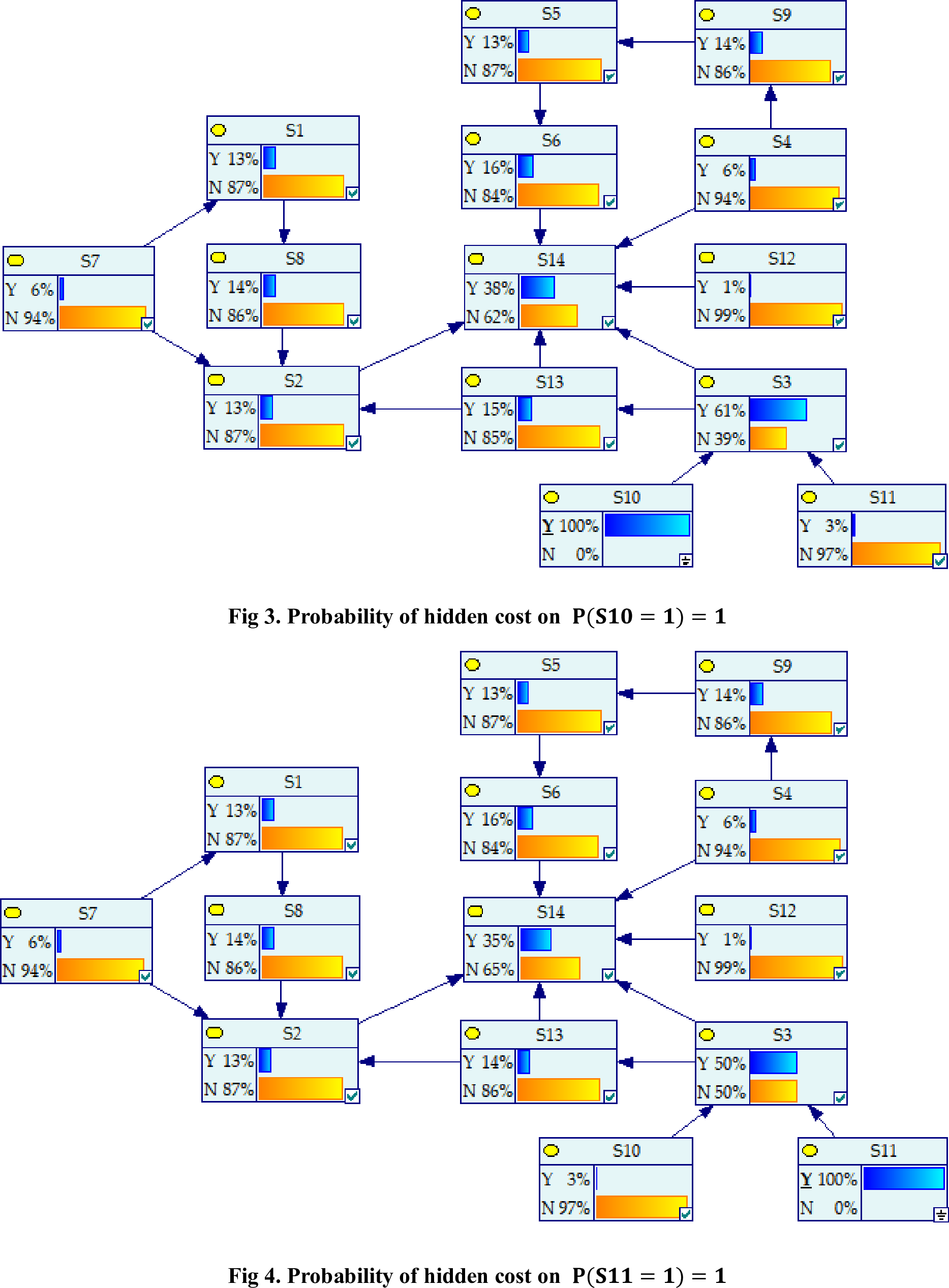

Supplement: S8 File — (ZIP) [file pone.0252138.s008.zip › S8 Data of the points extracted from images for analysis/S8 2.Data of the points extracted from images for analysis.tif]

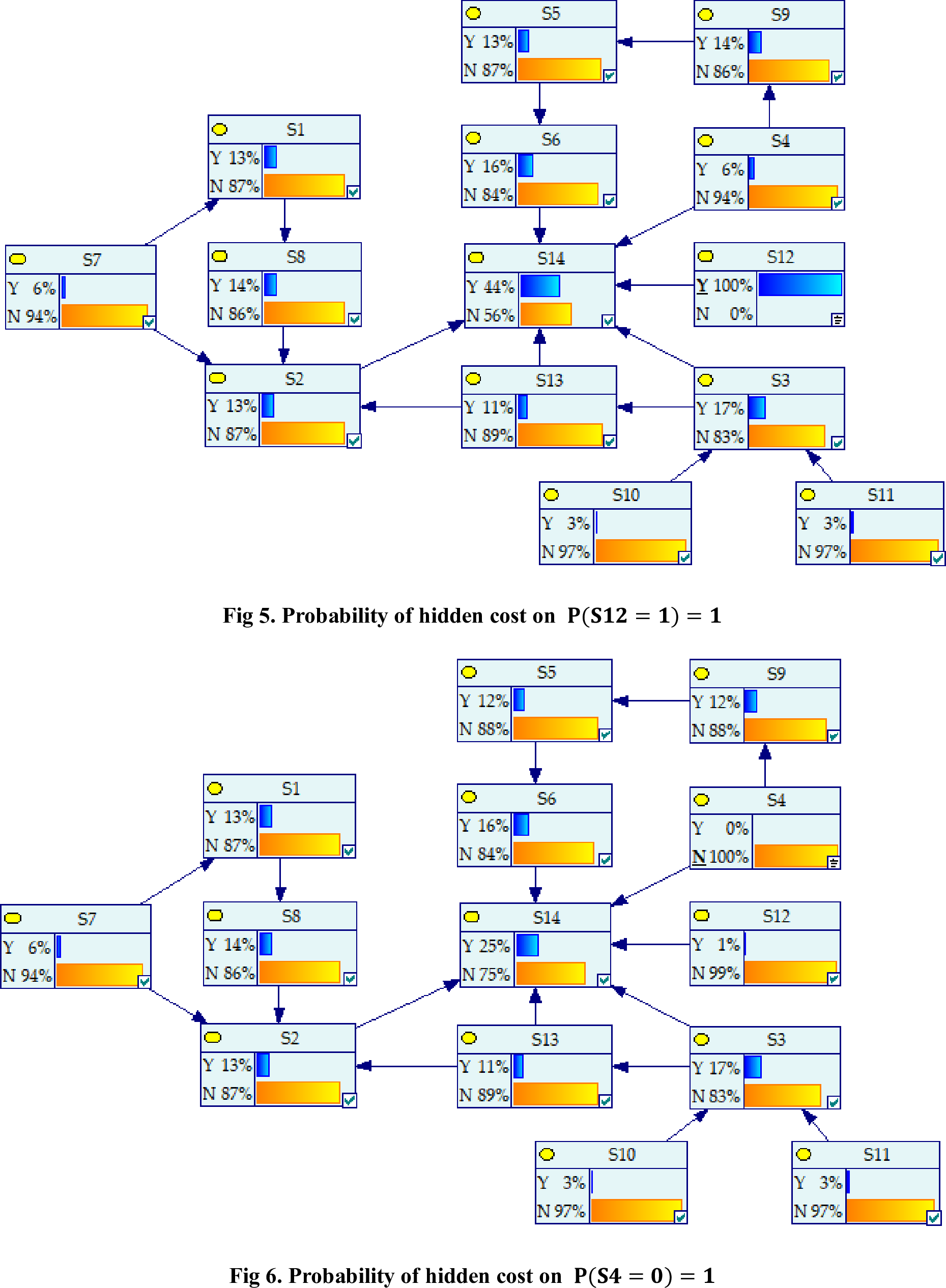

Supplement: S8 File — (ZIP) [file pone.0252138.s008.zip › S8 Data of the points extracted from images for analysis/S8 3.Data of the points extracted from images for analysis.tif]

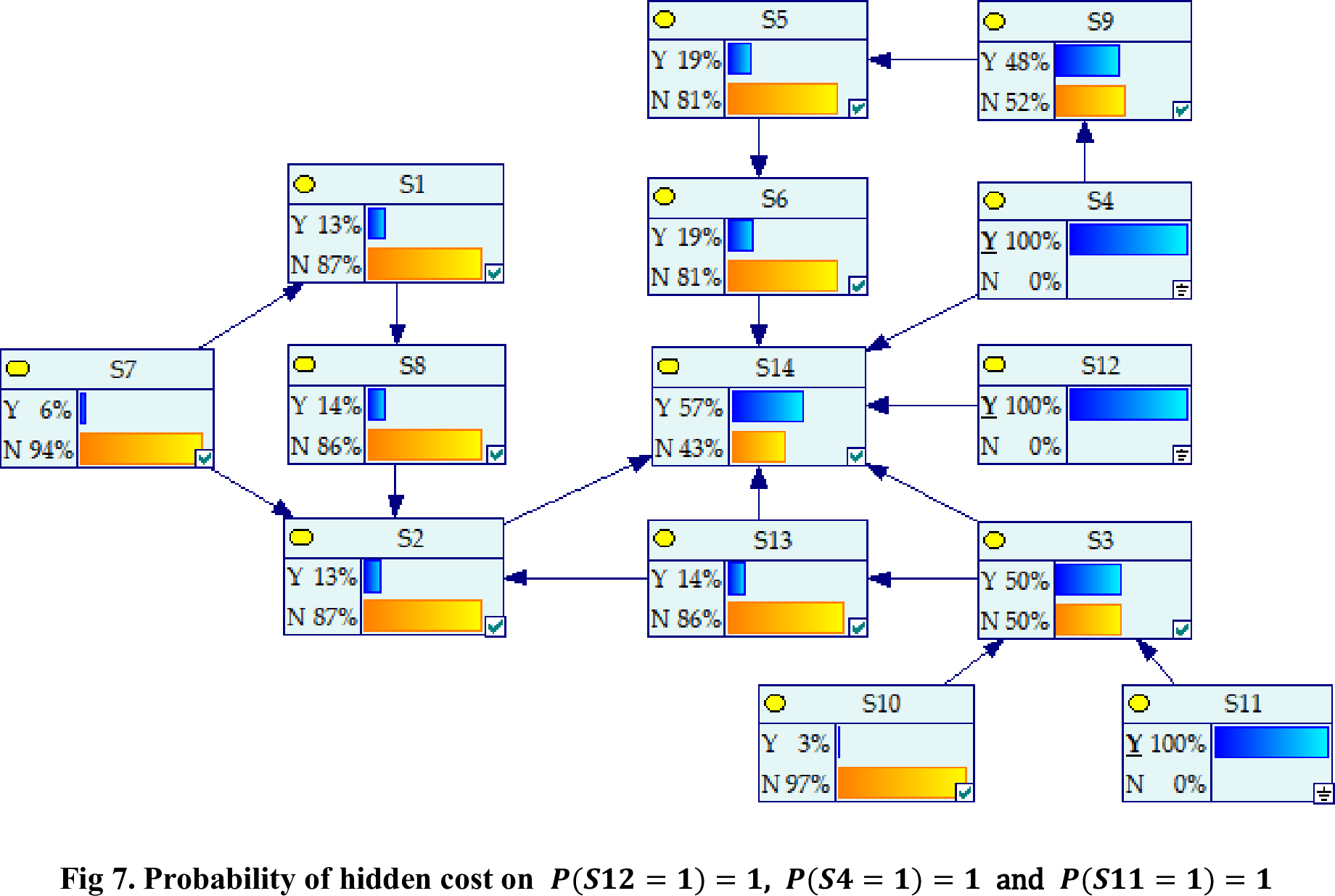

Supplement: S8 File — (ZIP) [file pone.0252138.s008.zip › S8 Data of the points extracted from images for analysis/S8 4.Data of the points extracted from images for analysis.tif]
